# Mastoparan, a Peptide Toxin from Wasp Venom Conjugated Fluvastatin Nanocomplex for Suppression of Lung Cancer Cell Growth

**DOI:** 10.3390/polym13234225

**Published:** 2021-12-02

**Authors:** Nabil A. Alhakamy, Osama A. A. Ahmed, Shadab Md, Usama A. Fahmy

**Affiliations:** 1 Department of Pharmaceutics, Faculty of Pharmacy, King Abdulaziz University, Jeddah 21589, Saudi Arabia; nalhakamy@kau.edu.sa (N.A.A.); osama712000@gmail.com (O.A.A.A.); shaque@kau.edu.sa (S.M.); 2 Center of Excellence for Drug Research and Pharmaceutical Industries, King Abdulaziz University, Jeddah 21589, Saudi Arabia; 3 Mohamed Saeed Tamer Chair for Pharmaceutical Industries, King Abdulaziz University, Jeddah 21589, Saudi Arabia

**Keywords:** mastoparan, cytotoxicity, fluvastatin, nanocomplex, lung cancer, peptide

## Abstract

Lung cancer has a very low survival rate, and non-small cell lung cancer comprises around 85% of all types of lung cancers. Fluvastatin (FLV) has demonstrated the apoptosis and suppression of tumor-cell proliferation against lung cancer cells in vitro. Drug–peptide nanoconjugates were found to enhance the cytotoxicity of anti-cancer drugs. Thus, the present study aimed to develop a nanocomplex of FLV with mastoparan (MAS), which is a peptide that has membranolytic anti-tumor activity. The nanocomplex of FLV and MAS (MAS-FLV-NC) was prepared and optimized for particle size using Box–Behnken design. The amount of FLV had the highest influence on particle size. While higher levels of FLV and incubation time favored higher particle size, a higher level of sonication time reduced the particle size of MAS-FLV-NC. The optimum formula of MAS-FLV-NC used 1.00 mg of FLV and was prepared with an incubation time of 12.1339 min and a sonication time of 6 min. The resultant particle size was 77.648 nm. The in vitro cell line studies of MAS-FLV-NC, FLV, and MAS were carried out in A549 cells. The IC_50_ values of MAS-FLV-NC, FLV, and MAS were 18.6 ± 0.9, 58.4 ± 2.8, and 34.3 ± 1.6 µg/mL respectively, showing the enhanced cytotoxicity of MAS-FLV-NC. The apoptotic activity showed that MAS-FLV-NC produced a higher percentage of cells in the late phase, showing a higher apoptotic activity than FLV and MAS. Furthermore, cell cycle arrest in S and Pre G1 phases by MAS-FLV-NC was observed in the cell cycle analysis by flow cytometry. The loss of mitochondrial membrane potential after MAS-FLV-NC treatment was significantly higher than those observed for FLV and MAS. The IL-1β, IL-6, and NF-kB expressions were inhibited, whereas TNF-α, caspase-3, and ROS expressions were enhanced by MAS-FLV-NC treatment. Furthermore, the expression levels of Bax, Bcl-2, and p53 strongly established the enhanced cytotoxic effect of MAS-FLV-NC. The results indicated that MAS-FLV-NC has better cytotoxicity than individual effects of MAS and FLV in A549 cells. Further pre-clinical and clinical studies are needed for developing MAS-FLV-NC to a clinically successful therapeutic approach against lung cancer.

## 1. Introduction

Lung cancer is a major concern due to its continuous rise in the last decade and has a very low survival rate. Lung cancer can be non-small cell lung cancer or small cell lung cancer. The former is the commonest type and comprises around 85% of all types of lung cancers [1]. While smoking cigarettes stands the highest risk factor, chronic lung diseases, environmental pollution, malnutrition, and genetic factors can also predispose to lung cancers [2]. The therapy of non-small cell lung cancer is presently through surgical procedures, radiation, or the use of chemotherapeutic agents. Meanwhile, adjuvant therapy is also useful [3].

The chemotherapy of lung cancer involves the use of one drug or a combination of drugs such as carboplatin, cisplatin, docetaxel, gemcitabine, nab-paclitaxel, paclitaxel, pemetrexed, and vinorelbine. The combined use of cisplatinum (or carboplatin) and paclitaxel (or any other similar anti-cancer agent) is also popular [3]. Interestingly, targeting of drugs to tumor cells by means of tumor-specific genes, proteins, or the tissue environment has emerged as promising approaches to reduce the systemic toxicity of chemotherapeutic agents. Meanwhile, targeting signaling pathways of tumor cells has also been proved successful in studies [4].

**Figure 1:**
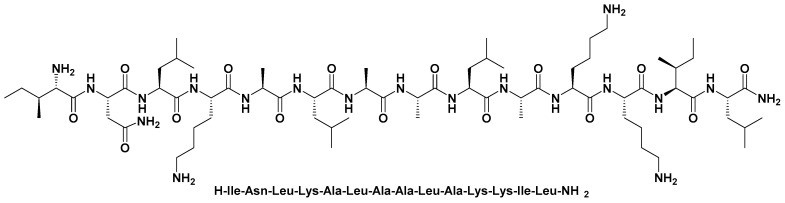
Chemical structure represents amino acids present in mastoparan.

The application of cell-penetrating peptides has been suggested to serve as transmembrane carriers for the purpose of tumor-targeted drug delivery. Furthermore, the modification, replacement, or reconstruction of the cell-penetrating peptides can be done for the purpose of improving the desired functionality [5]. Designed cell-penetrating peptides are an attractive strategy wherein hydrophilic and hydrophobic parts of different sources may be used. A combination of galanin and mastoparan (MAS) (known as transportan) is an example of such an approach. In this approach, the bioactive neuropeptide galanin is transported by conjugation with MAS [6]. MAS is a peptide found in wasp venom and has potent bioactivity [7]. As a peptide, MAS is composed of 14 amino acids (Figure 1) such as Ile-Asn-Leu-Lys-Ala-Leu-Ala-Ala-Leu-Ala-Lys-Lys-Ile-Leu-NH2 [8]. Thus, MAS is basically amphipathic in nature, but it contains 71% of hydrophobic residues. Generally, MAS shows its microbicidal mechanism against micro-organisms via membranolytic activity, which lyzed the cell membrane and increased the permeability that ultimately causes cell death. Furthermore, in the case of tumors, MAS intercepts the tumor growth via driving antiproliferative activity against tumorous cells [9]. Additionally, MAS was found to use the intrinsic mitochondrial pathway to induce apoptosis [10]. It had demonstrated enhancement in etoposide-induced tumor cell death in vitro [11]. Therefore, these advantages render MAS a superior candidate for its use in the preparation of nanocomplexes (NCs) with an anti-cancer agent, where MAS can amplify its combined anti-cancer drug’s activities by increasing drug deposition in the targeted area by accelerating its permeability across the cells.

NCs for cancer chemotherapy have emerged out to be promising and conducive for personalized medicine [12]. Interestingly, drug–polymer, drug–lipid, and drug–DNA NCs are reported for cancer chemotherapy [13,14,15,16]. Recently, stimuli-responsive NCs are also reported for the enhancement of cytotoxicity [17]. The success of these systems can depend either on the enhanced cytotoxic action of the NC or the release of the cytotoxic agent after reaching the target tumor cell. Importantly, the NCs have emerged as an answer to the failure of nanoparticles to show their efficacy at a clinical level and the huge concern of toxicity. Moreover, from a formulation point of view, the preparation of NCs would be more cost-effective and reproducible in large-scale production compared to the nanoparticles.

The selection of drugs to prepare the NC for lung-cancer chemotherapy warrants adherence to two major aspects. One is the chemical nature conducive for conjugation and another is the action against lung-tumor cells. Fluvastatin (FLV) is such a drug and has shown apoptosis and suppression of tumor cell proliferation [18]. In the case of non-small cell lung cancer, inhibition of 3-hydroxy-3-methylglutaryl coenzyme A reductase (HMGCR) by FLV has been demonstrated [19]. Furthermore, FLV prevents lung adenocarcinoma bone metastasis by promoting an autophagic mechanism [20]. Thus, FLV was considered a good candidate for conjugation with MAS for the therapeutic application against lung cancer. Furthermore, the conjugation of FLV to TAT peptide has been shown to enhance the apoptotic activity in HepG2 cells [21].

The particle size of NC would be critical in their effectiveness as is observed with nanoparticles [22]. The formulation and process factors of nanocomplexation of FLV and MAS could be influential on the particle size. The amount of FLV, incubation time, and sonication time can have a good impact on the final particle size. The optimization of these factors can be carried out most effectively using a design of experiments approach. This can help reach the optimum formula and process most effectively. Furthermore, this approach avoids the drawbacks of a one factor at a time approach [23].

Thus, the preparation and optimization of FLV and MAS NCs (MAS-FLV-NCs) were planned in the present using a Box–Behnken design for the optimization of FLV, incubation time, and sonication time. The possible minimum particle size of the MAS-FLV-NCs was chosen as the target response. The optimized MAS-FLV-NC was further evaluated for in vitro cell line studies in A549 cells for the anti-cancer effect in comparison to FLV and MAS.

## 2. Materials and Methods

### 2.1. Materials

Mastoparan was purchased from Sigma-Aldrich (St. Louis, MO, USA), while Fluvastatin was gifted from SPIMACO (Riyadh, Saudi Arabia). All other chemicals used in the study were of analytical reagent grade.

### 2.2. Formulation and Optimization of MAS-FLV Nanocomplex (MAS-FLV-NC)

MAS-FLV nanocomplex was prepared in accordance with the selected design. FLV and MAS were placed, in different proportions, in 20 mL of 0.01 M phosphate buffer bearing different pH levels before being whirled for a couple of minutes before its dissolution. Then, 1 mL aliquot of mixed solutions was diluted in 10 mL of the same buffer to determine the particle size and zeta potential. The design was generated and evaluated using Statgraphics software (Statgraphics Technologies, Inc., The Plains, VA, USA). FLV amount (mg, X1), incubation time (min, X2), and sonication time (min, X3) were considered as independent variables (Table 1) whereas particle size was considered as a dependent variable. The numerical optimization of the amount of FLV, incubation time, and sonication time were carried out by setting a minimum value for particle size as the goal.

### 2.3. In Vitro Cell Line Studies of MAS-FLV-NC in Lung Cancer Cells (A549 Cells)

#### 2.3.1. IC_50_ Determination

A549 cell lines were used to determine the IC_50_ of the FLV, MAS, and MAS-FLV-NC samples. Briefly, 5 × 10^3^ cells/well attached in a 96-well plate were taken for the study. After treatment with the samples corresponding to different concentrations of FLV for 4 h at 37 °C, the removal of supernatant was done, and 100 μL of DMSO was added. Afterward, the absorbance at 570 nm was noted using a microplate reader. Then, the IC_50_ values (*n* = 3) were calculated.

#### 2.3.2. Apoptotic Activity

Incubation of the A549 cells (96-well plate with a cell density of 1 × 10^5^ cells/well) with samples for 24 h was done for assessing the apoptotic activity of FLV, MAS, and MAS-FLV-NC samples [24]. After incubation, the centrifugation and separation of A549 cells were carried out. Later, these treated and separated cells were washed with phosphate-buffered saline. Subsequently, the cells were suspended in 500 μL of 1X binding buffer, stained using a commercially available kit (BD Bioscience, Stanford, CA, USA), and finally quantified by flow cytometry (FACS Calibur, BD Bioscience).

#### 2.3.3. Cell Cycle Analysis

Flow cytometry was done to assess cell cycle analysis. The procedure described for apoptotic activity was used for the cell cycle analysis, too.

#### 2.3.4. Mitochondrial Membrane Potential (MMP)

Briefly, to A549 cells (96-well plate with a cell density of 1.5 × 10^4^ cells/well), after incubation with FLV, MAS, and MAS-FLV-NC separately in 300 μL DMEM medium (supplemented with 10% FBS and 1% antibiotics), the probe (tetramethylrhodamine methyl ester) solution in the assay kit was added and incubated in the dark. Afterward, the addition of live-cell imaging buffer was done, and flow cytometry was carried out [25].

#### 2.3.5. Determination of Marker Molecules by ELISA

Interleukin-1β (IL-1β), tumor necrosis factor-alpha (TNF-α), caspase-3, reactive oxygen species (ROS), interleukin-6 (IL-6), and nuclear factor kappa B (NF-kB) were estimated to assess the cytotoxicity and apoptotic effect of samples. The analysis was done by ELISA kit for the biomarker (Invitrogen^®^, Thermo Fisher Scientific, Waltham, MA, USA). Briefly, A549 cells at a cell density of 5 × 10^4^ cells/well (96-well plate) were treated with FLV, MAS, and MAS-FLV-NC to equilibration. Later, 100 μL of the specific reagent was added, mixed for 30 s at 500 rpm, and kept aside for 30 min. Finally, IL-1β, TNF-α, caspase-3, ROS, IL-6, and NF-kB were estimated using the corresponding ELISA kit.

#### 2.3.6. Estimation of Bax, Bcl-2, and p53 Expressions Using RT-PCR

The levels of Bax, Bcl-2, and tumor protein P53 (p53) protein, after incubation with FLV, MAS, and MAS-FLV-NC separately, were determined by the RT-PCR method [26]. The Qiagen RNA/BioRad syber green PCR MMX kit and Rotorgene RT- PCR system (Rotor-Gene 1.7.87 software, Biorad, CA, USA) were used in the study.

## 3. Results

### 3.1. Formulation and Optimization of MAS-FLV-NC

MAS-FLV-NC was optimized by a three-factor, three-level BBD. The particle size values obtained for the design trials of MAS-FLV-NC are presented in Table 2. The predicted particle size values were well matching to the observed values and confirmed the validity of the design model.

The analysis of variance (ANOVA) data (Table 3) showed that Factor A (FLV), Factor B (incubation time), and Factor C (sonication time) had a significant influence on the particle size of MAS-FLV-NC. Furthermore, the interaction terms AA also had a significant influence on the particle size. The R-squared and the adjusted R-squared values were 96.0011 and 88.8032% respectively.

**Figure 2:**
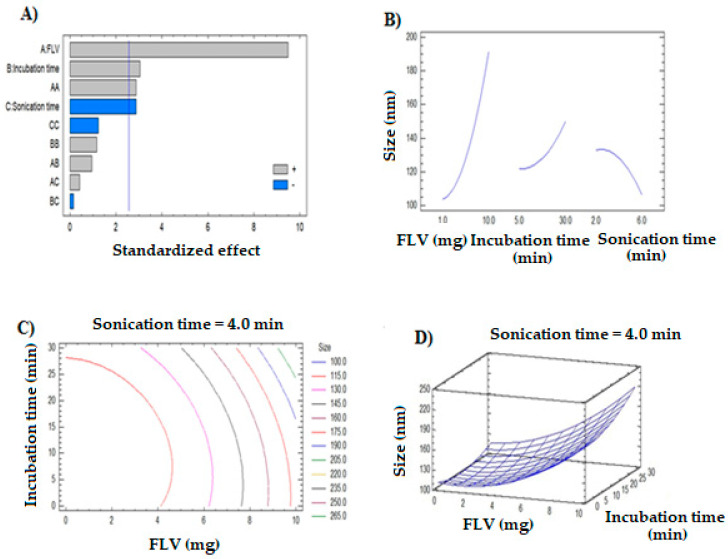
Plots represents the effects of dependent variables over particle size of MAS-FLV-NC: (**A**) Pareto chart, (**B**) main effects, (**C**) contour, (**D**) response surface.

The polynomial equation for the particle size is given in Equation (1). The coefficients for the independent factors implied that FLV had the highest influence since both Factors A and A^2^ had effects. The particle size was influenced by the factors in the order FLV > incubation time > sonication time. This was further confirmed by the Pareto chart (Figure 2A). The Pareto chart also confirmed the significant effects of Factors A, A^2^, B, and C. The Pareto chart showed positive effects for FLV, incubation time, and A^2^. Meanwhile, the negative effect of sonication time was shown by the Pareto chart. From these results, it can be derived that high levels of FLV and incubation time favor the high particle size of nanocomplex. Meanwhile, a high level of sonication time produces nanocomplexes with low particle sizes. This was further confirmed from the curves of individual factors shown in the main effects plot (Figure 2B). The interaction effects of the factors can be determined from the iso-value curves in the contour plot (Figure 2C) and the elevation of the response surface (Figure 2D).

Size = 106.997 − 4.11728 A − 1.11311 B + 9.33194 C + 0.975309 A^2^ + 0.111111 AB + 0.305556 AC + 0.0512 B^2^ − 0.04 BC − 2.125 C^2^ (1)

The optimum formula of MAS-FLV-NC obtained is provided in Table 4. The particle size response of the optimized MAS-FLV-NC formula was 77.648 nm.

### 3.2. In Vitro Cell Line Studies of MAS-FLV-NC in Lung Cancer Cells (A549 Cells)

#### 3.2.1. IC_50_ Determination

The IC_50_ values of FLV, MAS, and MAS-FLV-NC samples were studied by MTT assay in A549 cells. The IC_50_ values were found in the order FLV > MAS > MAS-FLV-NC. The IC_50_ value of MAS-FLV-NC (18.6 ± 0.9 µg/mL) was significantly (*p*-value < 0.05) less compared to those of FLV (58.4 ± 2.8 µg/mL) and MAS (34.3 ± 1.6 µg/mL).

#### 3.2.2. Apoptotic Activity

**Figure 3:**
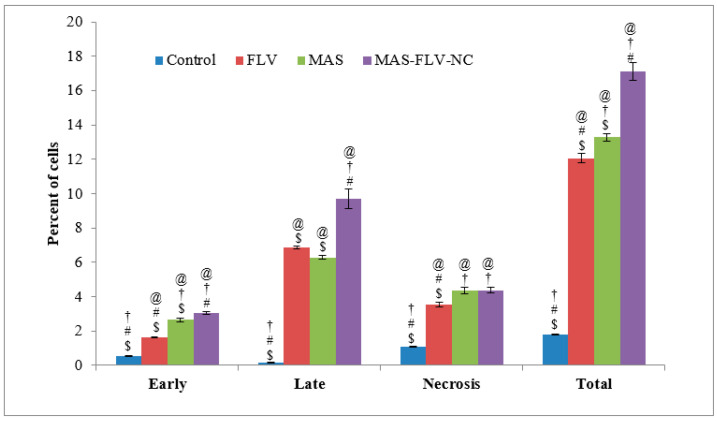
Comparative apoptotic effects of control, FLV, MAS, and MAS-FLV-NC samples on A549 cells in terms of percent cells observed (statistical inferences: @, *p* < 0.05, with respect to control; †, *p* < 0.05, with respect to FLV; #, *p* < 0.05, with respect to MAS; $, *p* < 0.05, with respect to MAS-FLV-NC).

The results of evaluation apoptotic activities (Figure 3) showed that the MAS-FLV-NC increased the cell percentage in both late-stage and total numbers. The late and total cell percentages of MAS-FLV-NC were statistically significant (*p*-value < 0.05) than those produced by FLV and MAS. The higher percent of cells in the late stage is a strong indication of the high apoptotic activity of the sample. MAS-FLV-NC showed 9.7 ± 0.57% cells in the late phase, whereas FLV and MAS showed 6.87 ± 0.07 and 6.28 ± 0.12%, respectively. Meanwhile, all the samples had a similar effect in the necrosis phase.

#### 3.2.3. Cell Cycle Analysis

**Figure 4:**
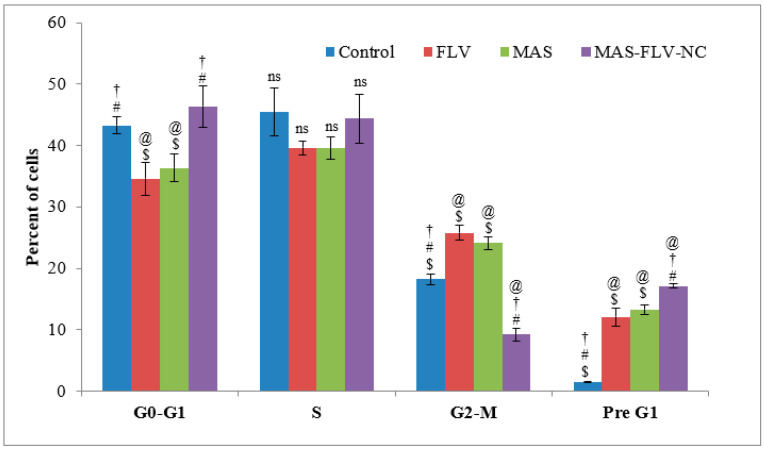
Comparative effects of control, FLV, MAS, and MAS-FLV-NC samples on the cell cycle of A549 cells in terms of percent cells (statistical inferences: @, *p* < 0.05, with respect to control; †, *p* < 0.05, with respect to FLV; #, *p* < 0.05, with respect to MAS; $, *p* < 0.05, with respect to MAS-FLV-NC; ns, *p* > 0.05, not significant with any group).

The cell cycle analysis (Figure 4) showed that MAS-FLV-NC had an effect in the Pre G1 phase, as indicated by the high percentage of cells and was significantly higher (*p*-value < 0.05) than those observed for FLV and MAS. Meanwhile, the effects of FLV and MAS in the Pre G1 phase and all other phases were similar (*p*-value > 0.05). Surprisingly, MAS-FLV-NC has the least percent of cells in the G2-M phase. This implies that MAS-FLV-NC induces cell cycle arrest at the S-phase.

#### 3.2.4. Mitochondrial Membrane Potential (MMP)

**Figure 5:**
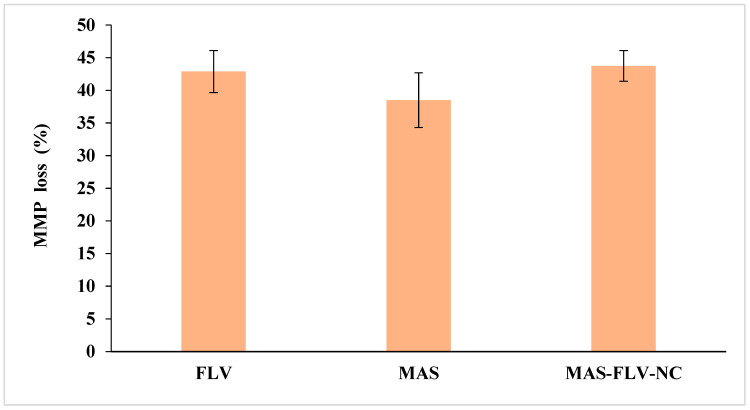
Comparative mitochondrial membrane potential (MMP) analysis results of control, FLV, MAS, and MAS-FLV-NC samples on A549 cells in terms of MMP loss (statistical inference: There were no significant differences between the sample treatments (*p* > 0.05)).

The MMP analysis was performed for determination of apoptotic activity, and the mean value of percent MMP loss was found to be highest for MAS-FLV-NC compared to FLV and MAS (Figure 5). However, there were no significant differences between the sample treatments (*p*-value > 0.05). FLV has proven effects on MMP, and the results suggested that the formation of NC maintained this effect [27].

#### 3.2.5. Determination of Marker Molecules by ELISA

**Figure 6:**
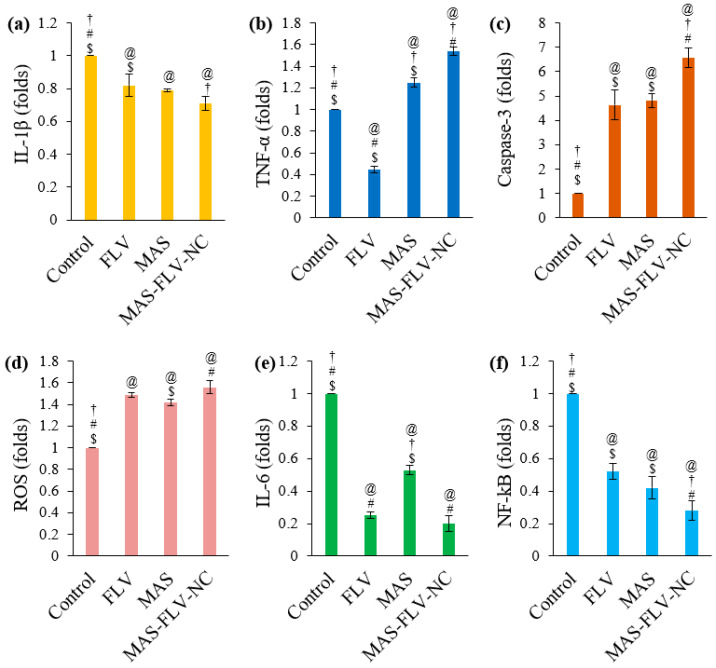
Comparative ELISA analysis results of control, FLV, MAS, and MAS-FLV-NC samples on A549 cells (**a**) IL-1β, (**b**) TNF-α, (**c**) Caspase-3, (**d**) ROS, (**e**) IL-6, (**f**) NF-kB (statistical inferences: @, *p* < 0.05, with respect to control; †, *p* < 0.05, with respect to FLV; #, *p* < 0.05, with respect to MAS; $, *p* < 0.05, with respect to MAS-FLV-NC).

The results are expressed as a fold change in comparison to the control sample of A549 cells without any treatment (Figure 6). The MAS-FLV-NC showed a significant (*p*-value < 0.05) decrease in the IL-1β level compared to control and FLV samples (Figure 6a). However, the reduction was not significant (*p*-value > 0.05) compared to that produced by MAS. Meanwhile, the highest level of TNF-α after treatment with MAS-FLV-NC also showed its high cytotoxicity (*p*-value < 0.05) than FLV and MAS (Figure 6b). Notably, FLV reduced the expression of TNF-α compared to the control. Higher levels of caspase-3 indicate higher cytotoxicity [28], and in the present study, the highest level of caspase-3 was produced by MAS-FLV-NC, showing its significantly higher (*p*-value < 0.05) cytotoxicity (Figure 6c). Meanwhile, there was a significant difference between the levels of caspase-3 produced by FLV and MAS (*p*-value > 0.05). However, FLV, MAS, and MAS-FLV-NC showed significantly higher (*p*-value < 0.05) cytotoxicity compared to the control. Results of antioxidant study in terms of ROS demonstrated the highest mean value of ROS for MAS-FLV-NC. However, there was no significant difference (*p*-value > 0.05) between the effects of FLV and MAS-FLV-NC. Nevertheless, FLV, MAS, and MAS-FLV-NC had a significant effect (*p*-value < 0.05) compared to control. In the case of IL-6, a reduction in the level was observed for MAS-FLV-NC (Figure 6e). Moreover, the reduction in IL-6 expression after MAS-FLV-NC treatment was statistically significant (*p*-value < 0.05) compared to control and MAS treatments but not to FLV treatment (*p*-value > 0.05). It has been already demonstrated in reported studies that both FLV and MAS reduce IL-6 expression [29,30]. Low levels of NF-kB suppress tumor progression and growth, and MAS-FLV-NC showed a significant reduction (*p*-value < 0.05) in the expression of NF-kB compared to all other samples (Figure 6f). The expression of NF-kB by FLV and MAS was similar (*p*-value > 0.05) but was significantly lower (*p*-value < 0.05) than the control treatment.

#### 3.2.6. Estimation of Bax, Bcl-2, and p53 Expressions Using RT-PCR

**Figure 7:**
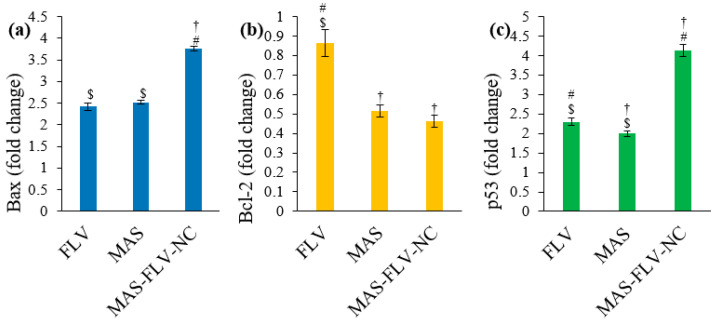
Results of comparative efficacy analysis of control, FLV, MAS, and MAS-FLV-NC samples on A549 cells (**a**) Bax, (**b**) Bcl-2, (**c**) p53 (statistical inferences: †, *p* < 0.05, with respect to FLV; #, *p* < 0.05, with respect to MAS; $, *p* < 0.05, with respect to MAS-FLV-NC).

The Bax expression was significantly higher (*p*-value < 0.05) for MAS-FLV-NC (Figure 7a). Thus, MAS-FLV-NC enhances the apoptosis potential of FLV and MAS in the form of NC. The Bax expression was similar for FLV and MAS (*p*-value > 0.05). Similar enhancement of apoptosis by MAS-FLV-NC through the reduced expression of Bcl-2 was also observed (Figure 7b). MAS-FLV-NC showed a significantly low level of Bcl-2 compared to that produced by FLV (*p*-value < 0.05) and comparable to that produced by MAS (*p*-value > 0.05). Meanwhile, MAS produced a significantly low level of Bcl-2 (*p*-value < 0.05) compared to FLV. Higher levels of p53 expression indicate higher levels of apoptosis and cytotoxicity. Thus, the significantly higher level (*p*-value < 0.05) of MAS-FLV-NC compared to FLV and MAS indicates its strong apoptosis activity (Figure 7c). Meanwhile, the expression of p53 was higher with FLV (*p*-value < 0.05) compared to that with MAS.

## 4. Discussion

### 4.1. Optimization and Selection of Best Possible MAS-FLV-NC

This study has been designed with novelty to improve the anti-lung cancer activity of FLV by conjugation with MAS. So, a NC-based nanoformulation of FLV and MAS was developed. In this case, for the selection of an optimized MAS-FLV-NC nanoformulation, a three-factor, three-level BBD design was used, where particle size was taken as a de-pendent variable [31]. Various formulations were prepared as per the software given runs, which characterized these nanoformulations on particle size, and we analyzed the effects of selected dependent variables over particle size by software. As a result, the software provided an optimized MAS-FLV-NC with a particle size of 77.648 nm. In the case of drug–carrier nanocomplexes, it is obvious that a higher amount of drug molecules would increase the chance of conjugation with the carrier molecule and therefore result in higher particle size. This might have resulted in the higher particle size of MAS-FLV-NC at higher FLV levels. This assumption has been confirmed by the previous study using a drug–lipid conjugate [32]. Similarly, the higher the incubation time of the drug with a carrier, there greater the chances of getting more drug molecules conjugated. This also can contribute to the larger particle size. Thus, the incubation time can be considered equivalent to the reaction time of the drug and carrier to form the nanocomplex, and the present observation can be justified [33]. In the case of sonication time, it is well established that sonication can be a means of the size reduction process by avoiding aggregation and providing proper distribution of nanostructure in the dispersion medium [34]. Thus, it is natural that higher sonication levels result in lower particle size. In addition, higher levels of sonication can reduce the chances of conjugation by impeding the contact of FLV and MAS molecules. The iso-value curves of the contour plot (Figure 2C) showed drastic shifting at higher values of FLV, indicating a higher influence of FLV on particle size than incubation time. This is due to the significant effect of interaction factor A^2^ in addition to Factor A. This was further confirmed from the significant increase in the elevation of the response surface of the particle size (Figure 2D) at higher FLV levels. The change in elevation of response surface on increasing the incubation time was not as high as that observed for FLV.

### 4.2. In Vitro Efficacy Studies of MAS-FLV-NC

To established the efficacy of optimized MAS-FLV-NC, various efficacy analysis was performed. In this case, initially, a comparative IC_50_ was determined between FLV, MAS, and MAS-FLV-NC samples. For this purpose, lung cancer cell line (A549 cells) was utilized. The outcomes demonstrated that the MAS-FLV-NC nanocomplex carried enhanced cytotoxicity compared to the individual effects of FLV and MAS. Such enhancement of cytotoxicity of nanocomplex has been observed with methotrexate when complexed with fullerenol [28]. Further apoptotic activity of MAS-FLV-NC was determined using flow cytometry [35]. The late and total cell percentages of MAS-FLV-NC treated cells were found to be more statistically significant (*p*-value < 0.05) than those produced by FLV and MAS. The higher percent of cells in the late stage is a strong indication of the high apoptotic activity of the MAS-FLV-NC. Simultaneously, cell cycle analysis showed that MAS-FLV-NC provoked cell cycle arrest in the S-phase. This was further confirmed from the higher percentage of cells in the S-phase for MAS-FLV-NC compared to those for FLV and MAS. Thus, ‘S-Phase block’ could be a mechanism of MAS-FLV-NC in exerting its cytotoxicity [36]. The loss of MMP could be used as a measure of the probability of apoptotic activity [37]. Therefore, the apoptotic activity of MAS-FLV-NC was further confirmed using MMP analysis by comparing with FLV and MAS (Figure 5), and MAS-FLV-NC exhibited more MMP loss than FLV and MAS, which confirmed the higher apoptotic activity of MAS-FLV-NC than FLV [18] and MAS alone.

The levels of molecular markers are important in the assessment of apoptosis and cytotoxicity. The effects of FLV, MAS, and MAS-FLV-NC samples on IL-1β, TNF-α, caspase-3, ROS, IL-6, and NF-kB were studied for the comparison of their apoptotic and cytotoxicity behavior [38]. The effect of samples to reduce the levels of IL-1β, which is a marker molecule favoring tumor cells, would be beneficial in the screening of cytotoxic activity [39]. The MAS-FLV-NC exhibited a decrease in the IL-1β level compared to control and FLV samples (Figure 6a). The inhibition of TNF-α by FLV is reported, and this might be the reason for such an observation [40]. Interestingly, MAS is reported to increase the expression of TNF-α [41]. While MAS has reported an action of enhanced expression of TNF-α, the higher expression level by MAS-FLV-NC shows that NC of MAS with FLV is even better in enhancing the TNF-α expression and cytotoxicity [42]. Thus, reducing IL-1β expression and increasing TNF-α level demonstrated amplified cytotoxicity against lung cancer cell lines. Higher levels of caspase-3 indicate higher cytotoxicity [28], and in the present study, the highest level of caspase-3 was produced by MAS-FLV-NC, showing its significantly higher (*p*-value < 0.05) cytotoxicity (Figure 6c). The ability of MAS to enhance the expression of caspase-3 is already established in a previous study [41]. Notably, MAS-FLV-NC produced an even higher expression of caspase-3 after the preparation of NC with FLV and favoring enhanced apoptosis. In addition, as observed with MAS-FLV-NC in the present study, a similar elevation of caspase-3 level on the conjugation of FLV with a TAT peptide is reported [21].

The enhancement of ROS by FLV via suppressing the mevalonate pathway is established [27]. Similarly, the enhancement of ROS by MAS via arachidonic cascade is also reported [43]. Therefore, it was expected that MAS-FLV-NC would produce a comparable or higher level of ROS than those produced by FLV and MAS. The result was as expected, and the MAS-FLV-NC sample showed the highest mean value of ROS (Figure 6d). Low concentrations of IL-6 enhance the cytotoxicity of TNF-α [44]. Thus, the result of IL-6 expression was confirmatory of the cytotoxicity of MAS-FLV-NC. Moreover, the NF-kB expression levels strongly supported the enhanced cytotoxicity of MAS-FLV-NC (Figure 6f).

Furthermore, the level of Bax, Bcl-2, and p53 expressions were estimated using RT-PCR [45]. The high Bax expression and reduced expression of Bcl-2 was observed for MAS-FLV-NC, which collectively enhanced the apoptosis potential of FLV and MAS when presented in the form of NC. Higher levels of p53 expression indicate higher levels of apoptosis and cytotoxicity. The results of p53 expression further confirmed the results of Bax and Bcl-2, showing that MAS-FLV-NC shows enhanced cytotoxicity compared to FLV and MAS. The ability of FLV to enhance p53 expression is already reported [20]. However, the MAS-FLV-NC was able to provide cytotoxicity more than that produced by FLV and MAS individually. Similar enhancement of apoptotic activity by changing the expressions of expression of Bax, Bcl-2, and p53 has been shown by reported nanoconjugates [46].

## 5. Conclusions

The NC of FLV and MAS was optimized for particle size with FLV, incubation time, and sonication time as independent factors. The optimum formula of MAS-FLV-NC had a particle size of 77.648 nm when prepared using 1.00 mg FLV, and with incubation and sonication times of 12.1339 and 6 min respectively. The in vitro cell line studies in A549 cells were carried out to assess the cytotoxicity of MAS-FLV-NC in comparison to FLV and MAS. In the MTT assay, MAS-FLV-NC showed an IC_50_ value of 18.6 ± 0.9 µg/mL, which was significantly less. The apoptosis study by flow cytometry showed that the late and total cell percentages of MAS-FLV-NC were higher than FLV and MAS treatments. Meanwhile, the cell cycle analysis showed that MAS-FLV-NC acts by cell cycle arrest in S and Pre G1 phases. Moreover, the percent MMP loss was also highest for MAS-FLV-NC. MAS-FLV-NC significantly inhibited IL-1β, IL-6, and NF-kB expressions in A549 cells. The expressions of TNF-α, caspase-3, and ROS were high in A549 cells after treatment with MAS-FLV-NC. All these suggested a significant cytotoxic effect of MAS-FLV-NC. Moreover, the expression levels of Bax, Bcl-2, and p53 strongly established the enhanced cytotoxic effect of MAS-FLV-NC. Thus, it can be concluded that the nanocomplexation of FLV and MAS results in a highly cytotoxic against A549 cells.

## Figures and Tables

**Table 1:** Formulation trials of MAS-FLV-NC for optimization.

Run	Factor Values		
	Factor A: FLV Amount (mg)	Factor B: Incubation Time (min)	Factor C: Sonication Time (min)
1	5.5	5	2
2	10	17.5	2
3	5.5	17.5	4
4	5.5	17.5	4
5	5.5	30	2
6	1	5	4
7	1	17.5	6
8	5.5	30	6
9	10	5	4
10	10	30	4
11	5.5	5	6
12	1	30	4
13	1	17.5	2
14	10	17.5	6
15	5.5	17.5	4

**Table 2:** The particle size values (observed and predicted) for various MAS-FLV-NC trials.

Run	Independent Factors			Dependent Factor	
	Factor A: FLV Amount (mg)	Factor B: Incubation Time (min)	Factor C: Sonication Time (min)	Response 1: Mean Particle Size (nm)	
				Observed	Predicted
1	5.5	5	2	132	125.8
2	10	17.5	2	191	193.9
3	5.5	17.5	4	129	128.0
4	5.5	17.5	4	127	128.0
5	5.5	30	2	145	156.0
6	1	5	4	90	103.9
7	1	17.5	6	82	79.1
8	5.5	30	6	121	127.3
9	10	5	4	176	179.4
10	10	30	4	234	220.1
11	5.5	5	6	112	101.0
12	1	30	4	123	119.6
13	1	17.5	2	119	111.4
14	10	17.5	6	165	172.6
15	5.5	17.5	4	128	128.0

**Table 3:** ANOVA data for particle size of MAS-FLV-NC formulations.

Source	Sum of Squares	Degrees of Freedom	Mean Square	F-Ratio	*p*-Value
A: FLV	15488.0	1	15488.0	89.76	0.0002
B: Incubation time	1596.13	1	1596.13	9.25	0.0287
C: Sonication time	1431.13	1	1431.13	8.29	0.0346
A^2^	1440.23	1	1440.23	8.35	0.0342
AB	156.25	1	156.25	0.91	0.3850
AC	30.25	1	30.25	0.18	0.6928
B^2^	236.308	1	236.308	1.37	0.2946
BC	4.0	1	4.0	0.02	0.8849
C^2^	266.769	1	266.769	1.55	0.2688
Total error	862.75	5	172.55	--	--
Total (corr.)	21574.9	14	--	--	--

**Table 4:** Optimum formula for the MAS-FLV-NC formulation.

Factor	Low	High	Optimum
FLV (mg)	1.0	10.0	1.00001
Incubation time (min)	5.0	30.0	12.1339
Sonication time (min)	2.0	6.0	6.0

## Data Availability

The data presented in this study are available in article.
